# Analysis of Mutation Spectra of 28 Pathogenic Genes Associated With Congenital Hypothyroidism in the Chinese Han Population

**DOI:** 10.3389/fendo.2021.695426

**Published:** 2021-07-02

**Authors:** Miao Huang, Xiyan Lu, Guoqing Dong, Jianxu Li, Chengcong Chen, Qiuxia Yu, Mingzhu Li, Yueyue Su

**Affiliations:** ^1^ Section of Endocrinology, Department of Pediatrics, Shenzhen Maternity and Child Healthcare Hospital, Southern Medical University, Shenzhen, China; ^2^ Department of Prenatal Diagnostic Center, Guangzhou Women and Children’s Medical Center, Guangzhou Medical University, Guangzhou, China

**Keywords:** congenital, hypothyroidism, targeted next-generation, sequencing, mutations, DUOX2, Chinese Han population

## Abstract

**Purpose:**

Congenital hypothyroidism (CH) is the most common neonatal endocrine disease; its early detection ensures successful treatment and prevents complications. However, its molecular etiology remains unclear.

**Methods:**

We used second-generation sequencing to detect 28 pathogenic genes in 15 Chinese Han patients with CH in Shenzhen, China, and analyzed the genetic pattern of the pathogenic genes through their pedigrees. The pathogenicity assessment of gene mutations was performed based on the American College of Medical Genetics and Genomics (ACMG) classification guidelines, inheritance models, and published evidence.

**Results:**

Mutations in several target genes were identified in 14 of 15 patients (93.33%); these mutations were distributed in eight genes (DUOX2, *DUOXA2, TPO, TG, TSHR, FOXE1, KDM6A*, and *POU1F1*). *DUOX2* exhibited the highest mutation frequency (44%, 11/25), followed by *TPO* (16%, 4/25) and TG (16%, 4/25). *DUOX2* exhibited the highest biallelic mutation (7/15). Eight out of 25 variants verified by the ACMG guidelines were classified as pathogenic (P, category 1) or possibly pathogenic (LP, Type 2), namely six variants of *DUOX2*, and one variant of *TPO* and *DUOXA2*. Five new mutations were detected: one in *DUOX2*, which was located in the splicing region of mRNA (c.1575-1G>A), three new missense mutants, p.A291T, p.R169W, and p. S1237dup, and one new *TPO* missense variant c.2012G>T (p.W671L). The main criteria for determining the genotype–phenotype relationship were a diagnostic detection rate of 53.33% (8/15) and combination of three or more gene mutations.

**Conclusions:**

CH gene mutations in the population may be mainly manifested in genes influencing thyroid hormone synthesis, such as *DUOX2* compound heterozygous mutations, which exhibited a high detection rate. The clinical manifestations are diverse, and mainly include transient CH. Therefore, genetic screening is recommended for CH patients to determine the correlation between clinical phenotypes and gene mutations, which will assist in clinical management.

## Introduction

The incidence of congenital hypothyroidism (CH) is 1/2,400 (0.042%), and it is the most commonly detected disease in Chinese newborn screenings (1); early diagnosis and treatment can prevent growth defects and mental retardation (2, 3). The etiology of CH is complicated and its pathogenesis remains unclear. Certain types of CH are familial, suggesting that its pathogenesis may be related to genetic factors (4, 5). Congenital hypothyroidism (CH) can be classified as permanent hypothyroidism (PCH) and transient congenital hypothyroidism (TCH) based on the treatment time. PCH requires permanent treatment, whereas TCH can be treated after a period of time. A study reported that the drug was discontinued after the treatment period (6). Current guidelines recommend that if the serum free T4 (fT4) concentration is reduced and thyroid-stimulating hormone (TSH) is significantly increased, levothyroxine (LT4) treatment should be started immediately (7). However, for CH with elevated serum TSH and normal fT4, the requirement for LT4 treatment remains controversial (8, 9). Currently, better methods to identify such42 conditions do not exist; thus, genetic screening may be helpful (10).

The synthesis, secretion, and action of thyroid hormones are regulated by various genes (11). The genes reported to affect thyroid hormone synthesis and secretion dysfunction comprise of *DUOX2, DUOXA2, IYD, SLC5A5, TG, TPO, SLC26A4*, and *SECISBP2* (12–15); genes that control the biosynthetic pathway of thyroid stimulating hormone include *TSH, THRB, TRHR*, and *IGSF1* (16); pituitary development-related genes *POU1F1, PROP1*, and *HESX1*, and genes that can cause diverse syndromes and abnormal thyroid function, including *GLIS3, GNAS, KDM6A, KMT2D, NKX2-1, NKX2-5*, and *UBR1* (17–19). All the above-mentioned gene mutations can lead to CH. It is important to determine53 whether these genetic mutations or mutation sites may help guide clinical treatment in children.

Most previous studies focus on a certain gene associated with CH (16), and there is a lack of joint screening studies for multiple genes, and related research on CH-related genes and clinical phenotypes. This study employed second-generation sequencing technology to detect 28 candidate genes related to CH, and combined information pertaining to family history and clinical phenotypes to clarify the etiology of CH.61. These results may help guide accurate clinical diagnosis and treatment, and reduce excessive medical treatment.

## Materials and Methods

### Patient Information

From September 2020 to March 2021, 15 CH cases were screened by genetic testing at the Shenzhen Maternal and Child Health Hospital after obtaining parental consent72. The age of the patients at the time of the study ranged from 3 months to 10 years.

The inclusion criteria were as follows: elevated TSH during neonatal screening, venous blood TSH serum level > 10 mIU/L using electrochemiluminescence assay (Cobas 6000, Roche Diagnostics) during follow-up, or CH manifestations (with persistent jaundice, growth and developmental delay, constipation, and feeding difficulties in children with abnormal TSH or thyroid hormone. Patients with acute infection. renal and liver dysfunction, 78 and glucocorticoid users were excluded from the study. When the TSH level was confirmed to be elevated (> 20 µIU/ml), replacement therapy with levothyroxine (LT4) was initiated. When the child turned 3 years old, oral thyroid hormone was stopped, and the thyroid hormone level was monitored regularly to determine whether the treatment should proceed. All patients underwent a thyroid ultrasound examination before treatment. The participants were all Chinese Hans, and the included patients comprised of eight females and seven males. Simultaneously, blood samples from the patient’s fathers and mothers were collected for genetic analysis. The parents of all participants signed an informed consent form. The study was approved by the Medical Ethics Committee of Shenzhen Maternal and Child Health Hospital and conducted in accordance with the Declaration of Helsinki.

### DNA Extraction and Sequencing

Peripheral blood was collected from the patients and their parents. Genomic DNA was prepared using a salting-out blood DNA extraction kit (Guangzhou Meji Biotechnology, China). The 28 potential pathogenic genes associated with congenital hypothyroidism, *DUOXA2* (NM_207581), *DUOX2* 94 (NM_014080), *FOXE1* (NM_004473), *GLIS3* (NM_152629), *GNAS* (NM_000516), *HESX1* 95 (NM_003865), *IGSF1* (NM_001170961), *IYD* (NM_203395), *KDM6A* (NM_021140), *KMT2D* 96 (NM_003482), *NKX2-1* (NM_001079668), *NKX2-5* (NM_004387), *PAX8* (NM_003466), *POU1F1* 97 (NM_000306), *PROP1* (NM_006261), *SEC077ISBP2* (NM_024_006261), *SLC16A2* (NM_006517), 98 *SLC26A4* (NM_000441), *SLC5A5* (NM_000453), *TG* (NM_003235), *THRA* (NM_199334), *THRB* 99 (NM_000461), *TPO* (NM_000547), *TRH* (NM_007117), *TRHR* (NM_003301, *TSHB* (NM_000549), *TSHR* (NM_000369), and *UBR1* (NM_174916), were selected for sequencing in the present study. The barcode library was established and the hybridization probe of the human exon library was used for hybridization capture using the NextSeq 500 platform (Illumina, San Diego, California, USA) for in-depth sequencing. This gene package-detection range included 28 related genes, 399 coding regions, and a total of 73,686 bases. The average coverage depth was 253 ± 68 ×, which was greater than 10 × coverage. There was a more than 20 × coverage area to account for 100% sequencing coverage. Simultaneously, we identified and analyzed the copy number variation of genes within the detection range.

### Analysis of Mutation Data

Sequencing results were analyzed using bioinformatics methods. The annotation range of the original second-generation sequencing data is the variation of each exon, variation in the 10 bp upstream and downstream of the exon, and known pathogenic mutations in the intron region. Variations included missense, nonsense, synonymous, frameshift, whole code, and cut. Quality control was performed on the variant data, and variants with a sequencing coverage depth < 20× were marked as low-quality variants. We performed a search of internal databases, dbSNP, ESP6500, gnomAD, and other population databases and marked SNPs and low-frequency benign variants. Prediction software was then used to predict if the mutations were conserved and the contribution of the mutations CH pathogenesis. We searched the HGMD, PubMed, Clinvar, and other databases and literature related to the variation, and referred to the American College of Medical Genetics and Genomics (ACMG) classification guidelines (20, 21) to classify the variation.

## Results

### Clinical Characteristics and Mutation Spectrum of Patients

**Figure 1:**
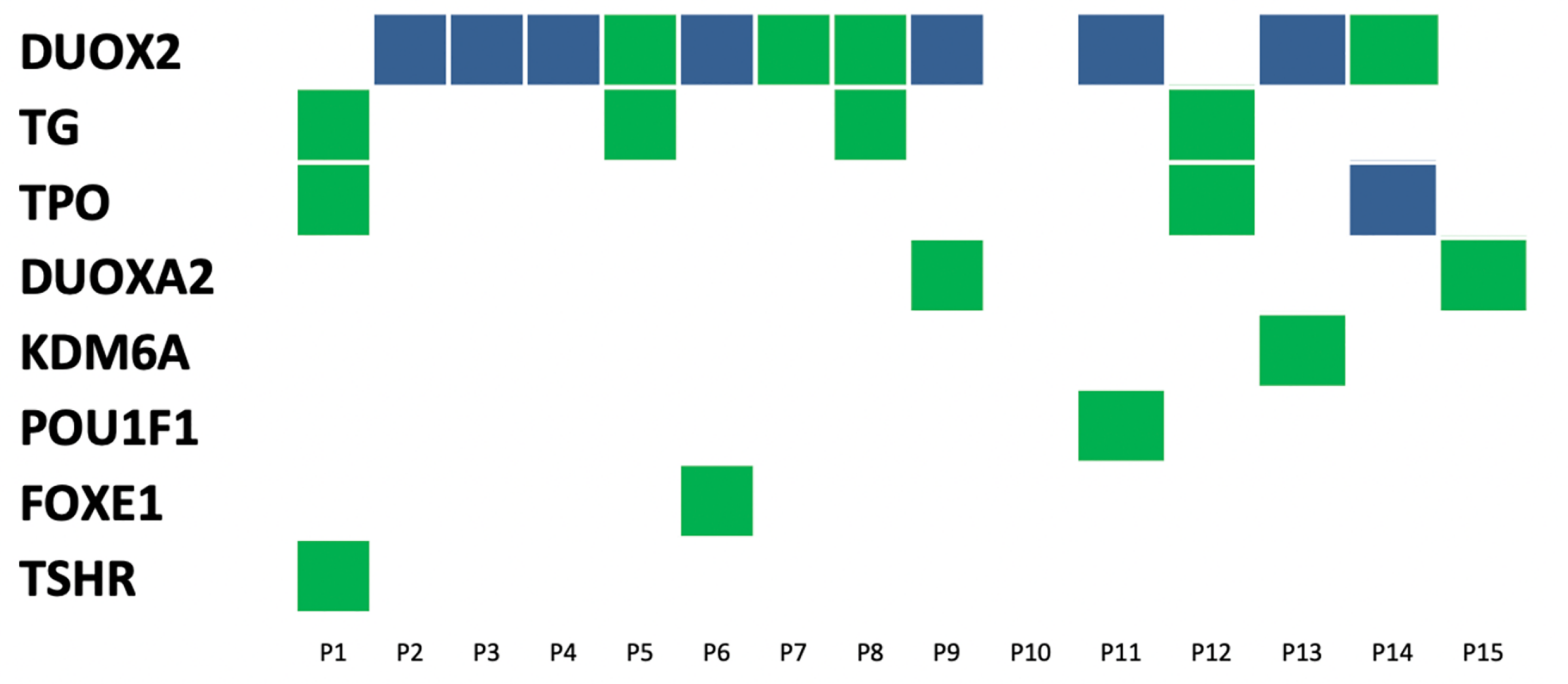
Distribution of variants in15 congenital hypothyroidism. The left side was 8 detected genes with variants, and the number on top of each box is the patient ID. Each column represents a patient and each row represents a gene. Blue blocks represent biallelic variants and green blocks represent monoallelic variants. Such as patient 9 carries variants on two genes: biallelic variants in the DUOX2 gene and monoallelic variant in DUOXA2.

This study included 15 unrelated patients diagnosed with CH with no history of thyroid disease, except for P2 and P3 patients. Ultrasonography of the thyroid revealed two cases of abnormal thyroid morphology — one case of thyroid hypoplasia and one case of ectopic thyroid. There were 13 cases of *in situ* glands (GIS), all of which were of normal sizes (**Table 1**). Fourteen out of 15 patients were identified as having a mutated gene (93.33%). Simultaneously, through copy number and SNP analysis, no variation in copy number potentially related to clinical manifestations was detected. The mutations were distributed among eight genes (*DUOX2, DUOXA2, TPO, TG, TSHR, FOXE1, KDM6A*, and *POU1F1*); the highest mutation frequency was detected in *DUOX2* (44%, 11/25), followed by *TPO* (16%, 4/25) and TG (16%, 4/25). Fourteen patients harbored mutations in at least one thyroid hormone-synthesizing gene (*DUOX2, DUOXA2, TPO, TG*, and *TSHR*). Twelve patients (80%, 12/15) possessed a combination of two gene locus mutations, and six patients (40%, 6/15) possessed a combination of 3 gene locus mutations. A total of 8 patients had double grades in *DUOX2* and *TPO*, and the highest gene mutation rate 127 was observed in *DUOX2* (7/15; see **Table 1** and **Figure 1**).

**Table 1:** Clinical Information, detected variants, and results of family segregation analysis of studied patients with CH.

Neonatal Period													
Patients ID	Sex	Birth weight(g)	Gestational age (week+ day)	Thyroid morphology	L-T4 (μg/kg/day) initial/current	Clinical phenotype	Development/age (y)	TSH (uIU/ml)	FT4 (pmol/l)	Detected variant	Father	Mother	Solved/ambiguous/unsolved
1	M	3000	39	N	8/0	TCH	Ht(-1	21.53	15.81	**TSHR**	NA	G/A	Ambiguous
							SD)/3			c.2272G>A(p.E75 8K)			
										**TG**c.958C>T			
										(p.R320C)			
										**TPO**c.2748+5_2748+6insG			
2*	F	3350	38	N	10/1	CH	N/2.3	35.65	8.75	**DUOX2c.505C>**	c.3709_3	c.505	Solved
										**T(p.R169W)/**	711dup	C>T	
										**c.3709_3711dup(p**			
										**.S1237dup)**			
3*	M	2850	37+3	N	10/0	TCH	N/4.1	56.47	6.45	**DUOX2**c.505C>T(p.R169W)/	c.3709_3711dup	c.505 C>T	Solved
										c.3709_3711dup(p			
										.S1237dup)			
4	F	1500	33+3	N	10/4	CH	Ht(-1	>100	3.31	**DUOX2**c.1588A>	c.1873C	c.158	Solved
							SD)/2			T(p.K530*)/	>T	8A>T	
										c.1873C>T(p.R625*)			
5	F	3100	39	undetected	7/2.5	CH	N/1.2	37.48	17.53	**DUOX2**c.2104_21	NA	NA	ambiguous
							5			06del(p.G702del)			
										**TG**			
										c.4859C>T(p.T162			
										0M)			
6	F	3200	39	N	10/2	PCH	Ht(-1	64.25	4.73	**DUOX2**c.1588A>	c.2654G	c.158	Solved
							SD)/3			T(p.K530*)/c.2654	>T	8A>T/	
										G>T(p.R885L)		G>C	
										**FOXE1**c.412G>C(p.E138Q)			
7	M	3500	40	N	10/0	TCH	N/3	32.83	13.1	**DUOX2**c.4408C>T(p.R14 70W)	C>T	NA	ambiguo us
8	M	3050	40	N	10/1	CH	N/2	>100	5.81	**DUOX2**	NA	G>A	ambiguous
										c.3329G>A(p.R11 10Q)			
										**TG**c.3035C>T(p.P 1012L)			
9	F	3900	40	N	10/5	CH	N/0.2	>100	6.37	**DUOX2**c.2654G>	c.3329G	c.265	Solved
										T(p.R885L)/	>A	4G>T	
										c.3329G>A(p.R11 10Q)			
										***DUOXA2***c.769+5G>A			
10	M	3024	39+6	The left lobe is absent	10/2.5	PCH	N/3.8	53.26	7.12	NA	–	–	unsolved
11	M	2790	40	N	10/8	CH	N/0.3	57.54	8.13	**DUOX2**c.2654G>	G>T	G>A	Solved
										T(p.R885L)/			
										**c.871G>A(p.A291**			
										**T)**			
										**POU1F1**c.302C>A			
										(p.T101N)			
12	F	2965	39+5	N	10/0	TCH	Ht(-1	63.79	4.22	**TG**	NA	NA	ambiguous
							SD)/3			c.7753C>T(p.R25 85W)			
										**TPO**c.1082G>T			
										(p.R361L)			
13	M	3300	40+1	N	10/1	PCH	N/10	83.43	3.12	**DUOX2c.1575-1**	G>A	c.332	Solved
										**G>A**/c.3329G>A(p		9	
										.R1110Q)		G>A	
										**KDM6A**			
										c.1835G>T			
										(p.R612L)			
14	M	3026	39+7	N	10/2.5	PCH	N/8.9	43.85	7.53	**TPO**c.2268dup(p. E757*)/**c.2012G> T(p.W671L) DUOX2**c.2635G> A(p.E879K)	NA	NA	Solved
15	F	2750	38+2	N	0/5	CH	N/0.3	479	0.37	**DUOXA2**c.738C>	NA	C>G	ambiguous

CH, congenital hypothyroidism; m, month; d, day; y, year; F, female; M, male; TSH, thyroid-stimulating hormone; FT4, free tetraiodothyronine; *, P2 and P3 are siblings of the same family; NA, data not available. PCH, permanent CH; TCH, transient CH; NC, not to time to make sure the clinical phenotype; NA, data not available; N, normal; age; the time for genetic testing.

Patients P3 and P2 were the children of a brother and sister of the same family. Two heterozygous variants of the DUOX2 gene, c.505C>T (p.R169W) and c.3709_3711dup (p.S1237dup), were detected. The verification experiment showed that these two variants were inherited from the candidate’s mother and father (both heterozygous). At the same time, no129 copy number variation potentially related to clinical manifestations was detected by copy number and SNP analysis. The clinical manifestations of the siblings included congenital hypothyroidism, but the parents of the candidates showed no corresponding clinical manifestations. The older brother displayed TCH. His131 treatment was discontinued at the age of 3, however he was short in height.

### Evaluation of the Pathogenicity of Mutant Genes

As shown in **Table 2**, 14 patients harbored a total of 25 gene mutation sites. According to ACMG guidelines, only variants classified as pathogenic or potentially pathogenic are considered to be determinants of “genetic134 diagnosis”. Of the 25 validated variants, eight were classified as pathogenic (P, category 1) or potentially pathogenic (LP, category 2), namely six *DUOX2* genes, and one *TPO* and *DUOXA2* gene. A total of 18 variants were classified as variants of uncertain importance (category 3). Among the five new variants, one was classified as potentially pathogenic (mRNA splicing region mutant c.1575-1G>A in *DUOX2*), and the others were classified as VUS.

**Table 2:** Potential pathological variants detected in the present study.

Gene	cDNA change	Amino Acids change	GnomAD east asian	Status^a^	ACMG classificationa
**DUOX2**	c.505C>T	p.R169W	0·002	Novel	VUS
**DUOX2**	c.3709_3711dup	p.S1237dup	0·002	Novel	VUS
DUOX2	c.1588A>T	p.K530*	0·009	Known	P
DUOX2	c.1873C>T	p.R625*	<0.001	Known	P
DUOX2	c.2104_2106del	p.G702del	<0.001	Known	VUS
DUOX2	c.2654G>T	p.R885L	<0.001	Known	LP
DUOX2	c.4408C>T	p.R1470W	0.002	Known	VUS
DUOX2	c.3329G>A	p.R1110Q	0.003	Known	LP
DUOX2	c.1575-1G>A	–	–	Novel	LP
DUOX2	c.871G>A	p.A291T	–	Novel	VUS
DUOX2	c.2635G>A	p.E879K	<0.001	Known	LP
TG	c.4859C>T	p.T1620M	0.016	Known	VUS
TG	c.3035C>T	p.P1012L	0.028	Known	VUS
TG	c.7753C>T	p.R2585W	0.005	Known	VUS
TG	c.958C>T	p.R320C	<0.001	Known	VUS
TPO	c.2268dup	p.E757*	0.002	Known	P
TPO	c.1082G>T	p.R361L	0.009	Known	VUS
TPO	c.2012G>T	p.W671L	<0.001	Novel	VUS
TPO	c.2748+5_2748+6insG	–	–	Known	VUS
DUOXA 2	c.769+5G>A	–	–	Known	VUS
DUOXA 2	c.738C>G	p.Y246*	0.002	Known	LP
KDM6A	c.1835G>T	p.R612L	–	Known	VUS
POU1F 1	c.302C>A	p.T101N	0.006	Known	VUS
FOXE1	c.412G>C	p.E138Q	–	Known	VUS
TSHR	c.2272G>A	p.E758K	<0.001	Known	VUS

P, pathogenic; LP, likely pathogenic; VUS, variants of uncertain significance. *Premature termination of encoded amino acid.

Eleven *DUOX2* mutants were found in 11 individuals (**Table 2**). The detected *DUOX2* mutants included seven previously reported missense mutants, (p.K530*), (p.R625*), (p. G702del), (p.R885L), (p.R1470W), (p.R1110Q), and (p.E879K), a new mutant located in the splicing region of mRNA (c.1575-1G>A), and three new missense mutants p.A291T, p.R169W, and p. S1237dup. Among them, p.R169W and p.S1237dup were detected in siblings from the same family and from the mother and father, respectively, and none of them have been reported in related clinical cases. A new missense variant, c.2012G>T (p.W671L), was found to be associated with *TPO*.

### The Relationship Between Genotype and Phenotype

Through family verification and pathogenicity assessment, seven cases (patients 2, 3, 4, 6, 9, 11, 13) were considered “resolved”, and the diagnostic detection rate was 46.67% (**Table 1**). These “resolved” cases refer to the presence of at least two pathogenic variants in the same gene. These variants are from paternal or maternal lines, but not from a single parent. Due to the weak association between genotype and phenotype, a total of six cases (40%) were considered “ambiguous”. Moreover, one case of P10 was considered “unresolved” because it did not carry any genetic mutations, but a color Doppler ultrasound revealed hypoplasia of the left lobe. Among the resolved cases, three were monogenic, whereas four were polygenic. The distribution of genes in all the resolved cases showed a *DUOX2* heterozygous mutation. Notably, all “resolved” cases included patients with normal thyroid morphology. Therefore, the155 diagnosis rate of patients with normal thyroid morphology was 53.84% (**Table 1**).

In these “resolved” cases, oral eumethal therapy for patients P2, P3, P4, and P6 could not be stopped at 2 years of age, but the oral LT4 level was < 3 Ug/Kg·d. Patient P13 is now 10 years old. Genetic screening revealed a heterozygous mutation in *DUOX2* in this patient, which manifested as PCH, but was maintained by a low 157 dose of T4 (1 Ug/Kg·d). The patient’s height and intelligence were normal.

## Discussion

Second-generation sequencing was used to screen 28 pathogenic genes associated with CH. The screening showed that the gene mutation detection rate was 93.33%, and was mainly detected in genes that affect thyroid hormone synthesis, such as *DUOX2*, *TPO*, and *TG*. Previous studies have shown that the detection rate of *DUOX2* mutations in children with CH in China is as high as 28%-44%, whereas the detection rate obtained in this study was 44%, suggesting that *DUOX2* mutations may be the160 main cause of CH in the Chinese population (22–24). *DUOX2* is mainly involved in the production of peroxide protein complexes and catalyzes the synthesis of thyroid hormones in thyroid follicular cells (25). Studies have found that mutations in *DUOX2* can initiate CH, but the clinical phenotype, primarily the manifestation of TCH, is variable (26, 27). In this study, except for the mutations observed in patients P1, P10, and P12, all genes merged with one or two *DUOX2* mutations. These children exhibited various clinical manifestations, including severe CH, mild CH, hyper-TSH-emia, and permanent CH (28). In the resolved cases, compound heterozygous mutations in *DUOX2* were combined, and the oral LT4 dosage at the age of 2 years was less than 3 Ug/Kg·d. It is suggested that CH combined with compound heterozygous mutations of *DUOX2* is primarily the outcome of TCH. The small sample size in this study requires the165 correlation between gene mutations and clinical phenotypes to be further confirmed by studies with a larger sample size.

*TPO* encodes thyroid peroxidase (TPO), which catalyzes a key reaction in the synthesis of thyroid hormones and is inherited in an autosomal recessive manner (29). In this study, we detected a heterozygous mutation of P1 and P12 combined with the *TPO* gene, which was manifested as TCH. P14 combined with the *TPO* gene c.2012G>T (p.W671L) and c.2268dup (p.E757*) compound heterozygous mutations manifested as PTH at 10 years of age, wherein a thyroid color Doppler ultrasound171 indicated mild goiter. The rare missense mutation in *TPO* c.2012G>T (p.W671L) has not been reported in related clinical cases, but numerous clinical cases related to thyroid hormone production disorders have reported a rare variant of *TPO* c.2268dup (p.E757*). It is a homozygous mutation or forms a compound heterozygous state with other variants. Here, the patient’s 174 clinic al manifestations were as follows: the level of TSH increased in the neonatal period, that of thyroid hormone decreased, and goiter occurred during adolescence (13, 30). Moreover, functional experiments have shown that this mutation can cause reduced expression of176 TPO and loss of enzyme activity (31).

Zamproni (32) reported that a Chinese patient born to parents of unrelated descent was homozygous for a nonsense mutation (p.Y246X) and produced a truncated DUOXA2 protein with a missing transmembrane helix 5 and C-terminal cytoplasmic domains. DUOX2 cannot be recombined *in vitro*, thereby affecting the synthesis of thyroid hormones. Patient P15 harbored only one mutation in DUOXA2c.738C>G (p.Y246*), which originated from a mother with a normal phenotype. The clinical manifestations of TSH screening at birth were normal. Due to repeated jaundice, the altered blood thyroid hormone [TSH 479 (uIU/ml) and fT4 0.37 (pmol/l)] was associated with abdominal distension, constipation, low crying, weight loss, and other severe hypothyroidism. Patient P7 only revealed that *DUOX2* c.4408C>T (p.R1470W) harbored a gene mutation from a father with a normal phenotype, showing temporary hyper-TSH-emia, and the drug was discontinued at the age of 3. This rare variant has been reported in disorders of thyroid hormone synthesis, and the patient harbored a compound heterozygous mutation (33). The above two cases do not conform to the rules of Mendelian inheritance. We speculated that these cases were caused by other genes, which are excluded from the scope of this analyses, or there may be other genes that function in collaboration with these mutations to cause the disease, which requires further181 follow-up functional studies.

Herein, 8 of the 25 variants verified based on the ACMG guidelines were classified as pathogenic (P,183 category 1) or potentially pathogenic (LP, category 2), namely six variants of *DUOX2* (p.K530 *, p.R625*, p.R885L, p.R1110Q, c.1575-1G>A, p.E879K), and one variant of *TPO* (p.E757*) and *DUOXA2* (p.Y246*). These results indicated that that *DUOX2* mutations are the main pathogenic genes associated with CH in the Han population in China. Five new mutations were found, one *DUOX2* mutation, which was located in the splicing region of mRNA (c.1575-1G>A), three new missense mutants, p.A291T, p.R169W, and p.S1237dup, and one new *TPO* missense variant c.2012G>T (p.W671L). To date, these variants exhibit a low frequency in the reference population gene database. The region where the mutation is located is an important part of this protein, and the amino acid sequences of different species are highly conserved. Computer-aided analysis predicts that this mutation is more likely to affect protein structure and function. In this study, patients P2 and P3 represented two siblings. New rare variants that may be related to clinical manifestations were detected, which included two heterozygous variants of *DUOX2*, c.505C>T (p.R169W) and c.3709_3711dup (p.S1237dup). Family verification showed that these two variants were inherited from the mother and father of the candidate (both are heterozygous). The clinical manifestations included high TSH and low FT4 levels after birth. For the elder brother, thyroid hormone therapy can only be stopped at 4 years of age, whereas the younger sister is currently 2 years old and still requires oral thyroid hormone therapy. These two rare variants, c.505C>T and (p.R169W), have not been reported in relevant242 clinical cases. However, their relevance requires further clarification.

This study has some limitations. First, the sample size was relatively small; most of the patients had a short follow-up period, and the clinical phenotype data were not comprehensive. Therefore, it is not analytically conducive to determine the correlation between the detected mutation and clinical phenotype. Simultaneously, there is a lack of *in vitro* functional studies of new variants identified in the current study.

In summary, the mutation profiles of 15 Han patients with CH in Shenzhen, China were determined using second-generation sequencing targeting 28 known disease-causing genes. It is mainly manifested in genes that affect thyroid hormone synthesis—primarily compound heterozygous mutations in253 *DUOX2*. Moreover, the gene mutation detection rate was as high as 93.33%, and the new gene mutation rate was 5/25 (20%). Therefore, pathogenic gene screening for patients with CH, enriching the mutation spectrum, and identifying the relationship between gene mutations and phenotypes are the recommended steps to achieve accurate clinical diagnosis256 and treatment of CH.

## Data Availability Statement

The variation data reported in this paper have been deposited in the Genome Variation Map (GVM) [1] in Big Data Center [2], Beijing Institute of Genomics (BIG), Chinese Academy of Science, under accession numbers GVM000180 at http://bigd.big.ac.cn/gvm/getProjectDetail?project=GVM000180.

## Ethics Statement

The studies involving human participants were reviewed and approved by the Medical Ethics Committee of Shenzhen Maternal and Child Health Hospital. Written informed consent to participate in this study was provided by the participants’ legal guardian/next of kin.

## Author Contributions

MH and XL conceptualized and designed the study, and drafted the initial manuscript. GD conceptualized and designed the study. JL and CC performed the initial analyses, reviewed and revised the manuscript, and approved the final manuscript. QY performed the initial analyses and approved the final manuscript. ML and YS coordinated and supervised data collection, critically reviewed the manuscript and approved the final manuscript. All authors contributed to the article and approved the submitted version.

## Funding

This study was supported by Sanming Project of Medicine in Shenzhen (SZSM201812056).

## Conflict of Interest

The authors declare that the research was conducted in the absence of any commercial or financial relationships that could be construed as a potential conflict of interest.
